# Associations between genomic aberrations, increased nuchal translucency, and pregnancy outcomes: a comprehensive analysis of 2,272 singleton pregnancies in women under 35

**DOI:** 10.3389/fmed.2024.1376319

**Published:** 2024-04-03

**Authors:** Jia Huang, Dong Wu, Jia-Huan He, Jing-Yuan Wang, Xi Li, Zheng-Yuan Wang, Yue Wang, Hong-Yan Liu

**Affiliations:** ^1^Department of Medical Genetics, Henan Provincial People's Hospital, People's Hospital of Zhengzhou University, People's Hospital of Henan University, Zhengzhou, Henan, China; ^2^Department of Gynaecology and Obstetrics, Henan Provincial People's Hospital, People's Hospital of Zhengzhou University, People’s Hospital of Henan University, Zhengzhou, Henan, China

**Keywords:** increased nuchal translucency, copy number variation sequencing, chromosomal microarray, genomic aberrations, pregnancy outcomes

## Abstract

**Objectives:**

Regarding increased nuchal translucency (NT), the cutoff values used are heterogeneous in clinical practice, this study aims to assess the efficacy of prenatal detection for chromosomal abnormalities and pregnancy outcomes in fetuses with varying NT thicknesses, in order to provide data that supports informed prenatal diagnosis and genetic counseling for such cases.

**Methods:**

We included 2,272 pregnant women under 35 with singleton pregnancies who underwent invasive prenatal diagnosis between 2014 and 2022. The cohort comprised 2,010 fetuses with increased NT (≥2.5 mm) and 262 fetuses with normal NT but exhibiting a single soft marker. Prenatal diagnoses were supported by chromosomal microarray (CMA) and copy number variation sequencing (CNV-seq) analyses.

**Results:**

The detection rates of numerical chromosomal abnormalities were 15.4% (309/2,010) and 17.3% (297/1,717) in the NT ≥2.5 and ≥ 3.0 groups, respectively. Pathogenic/likely pathogenic CNV incidence increased with NT thickness (χ2 = 8.60, *p* < 0.05), peaking at 8.7% (22/254) in the NT 4.5–5.4 mm group. Structural defects were found in 18.4% of fetuses with NT values between 2.5 mm and 2.9 mm. Chromosomal abnormality rates in the isolated increased NT groups of 2.5–2.9 mm and 3.0–3.4 mm were 6.7% (16/239) and 10.0% (47/470), respectively, with no statistical significance (χ2 = 2.14, *p* > 0.05). Fetuses with NT thickness between 2.5 and 2.9 mm combined with the presence of soft markers or non-lethal structural abnormalities exhibited a significantly higher chromosomal abnormality risk (19.0%) compared to fetuses with isolated increased NT ranging from 3.5 to 4.4 mm (13.0%). Pregnancy termination rates increased with NT thickness (χ2 = 435.18, *p* < 0.0001), ranging from 12.0% (30/249) in the NT 2.5–2.9 mm group to 87.0% (141/162) in the NT ≥ 6.5 mm group.

**Conclusion:**

CMA or CNV-seq exhibited good performance in identifying genomic aberrations in pregnancies with increased NT thickness. NT ranging from 2.5 mm to 2.9 mm elevated the risk of fetal chromosomal abnormalities, particularly when combined with other soft markers.

## Introduction

Nuchal translucency (NT), measuring the amount of fluid behind the fetal neck in the first trimester of pregnancy, is a crucial indicator for assessing fetal abnormalities in prenatal screening. The Society of Obstetricians and Gynecologists of Canada (SOGC) and the Canadian College of Medical Geneticists (CCMG) propose a 3.5 mm threshold for guiding invasive prenatal diagnosis (1). While, the American College of Obstetricians and Gynecologists (ACOG) suggests a threshold of 3.0 mm or exceeding the 99th percentile of the crown-rump length for elevated risk (2). Others advocate adopting a cutoff at the 99th percentile, which is superior to the fixed cutoff of 3.0 mm for the identification of atypical chromosome abnormalities (3). However, numerous studies propose a cutoff value of 2.5 mm for heightened risk (4–6). This lack of standardization and consensus generates confusion in clinical practice, particularly for fetuses falling within the ambiguous zone of NT ranging from 2.5 to 2.9 mm, complicating laboratory interpretations.

There is increasing evidence suggesting a potential increase in the risk of fetal abnormalities when fetal nuchal translucency thickness falls within the range of 2.5 mm to 2.9 mm (7–9). However, the limited number of cases within this NT range and the widely varying risk values pose challenges. The question arises: Should non-invasive prenatal testing (NIPT) or invasive chorionic villus sampling (CVS) be recommended for these pregnant women? Alternatively, could NT thickness be employed more effectively when combined with other markers? Given the age-related increase in the incidence of fetal chromosomal abnormalities, this study exclusively focused on pregnant women under 35 years old to minimize age-related confounding factors. The primary aim of this research was to investigate the risk of chromosomal diseases and submicroscopic pathogenic copy number variations in fetuses with increased NT, with emphasis on NT measurements between 2.5 mm and 2.9 mm. Importantly, we sought to establish a correlation between NT thickness and pregnancy outcomes through follow-up assessments, providing data for genetic counseling and aiding in the selection of appropriate prenatal diagnostic methods during pregnancy.

## Methods

### Patient enrollment

This retrospective study included 2,010 pregnant women under 35 years old (mean age 27.3 ± 3.9 years) with singleton pregnancies enrolled at the prenatal diagnosis center of Henan Provincial People’s Hospital from 2014 to 2022. All participants underwent first-trimester ultrasonography, revealing fetal NT thickness ≥ 2.5 mm between 11 to 13^+6^ gestational weeks. Concurrently, 262 age-matched controls (mean age 28.2 ± 3.8 years) with singleton pregnancies and NT thickness < 2.5 mm were recruited. Control group participants exhibited single soft markers on ultrasonography, including cases of choroid plexus cyst (*n* = 161), echogenic intracardiac focus (*n* = 49), single umbilical artery (*n* = 38), and mild pelvicalyceal separation (*n* = 14). Prenatal diagnostic samples comprised 1,410 CVS and 862 amniotic fluid samples.

### Clinical data collection and grouping

The pregnant women’s age, gestational age, and ultrasound examination data were extracted from the outpatient medical record system. The cohort was categorized into seven groups based on NT measurements: NT < 2.5 mm, NT 2.5–2.9 mm, NT 3.0–3.4 mm, NT 3.5–4.4 mm, NT 4.5–5.4 mm, NT 5.5–6.4 mm, and NT ≥ 6.5 mm. Additional ultrasound anomalies were identified during either the first or second trimester ultrasound scan before genetic testing, and ultrasound detection results led to stratification into groups: normal NT combined with a single soft mark (Group A), isolated increased NT (Group B), increased NT combined with soft mark/non-lethal structural abnormality (Group C) and increased NT combined with lethal structural abnormalities (Group D). Pregnancy outcomes and infant development were assessed using the Maternal and Child Health Registration System of Henan Province and through telephone interviews.

### Ethical statement

This study received ethical approval from the Ethics Committee of Henan Provincial People’s Hospital (2021-No. 171). Prior to participation, all individuals involved provided informed consent and underwent genetic counseling.

### Copy number variation analysis

Copy number variation analysis was conducted using a chromosomal array (CMA) with the SurePrint G3 ISCA V2 Human CGH Array Kit, 8x60K (Agilent, United States). Additionally, copy number variation sequencing was performed using the CNV-seq kit (Berry Genomics, China), and sequencing runs were carried out on the Illumina NextSeq500 platform (Illumina, United States). The sequencing data had a read length of 37 bp, with an average coverage of 0.1X. To assess the pathogenicity of variants, the American College of Medical Genetics and Genomics (ACMG) recommendations (10) were applied. The following resources were referenced for variant interpretation: Online Mendelian Inheritance in Man (OMIM) at https://omim.org, the Database of Chromosomal Imbalance and Phenotype in Humans using Ensembl Resources (DECIPHER) at https://decipher.sanger.ac.uk, the Database of Genomic Variants (DGV) at http://dgv.tcag.ca/dgv/app/home, the Human Gene Mutation Database (HGMD) at www.hgmd.cf.ac.uk/ac/index.php, ClinVar at www.ncbi.nlm.nih.gov/clinvar, and the Clinical Genome Resource Genome Dosage Map at https://search.clinicalgenome.org.

### Statistical analysis

Statistical analysis was conducted using SPSS 26 software. Normally distributed data were presented as means ± SD, while non-normally distributed data were expressed as medians. The Chi-square and trend Chi-square tests were employed to evaluate significant differences between groups. A two-sided *p* < 0.05 was considered statistically significant.

## Results

### Ultrasound results of fetuses in NT increased group

As shown in Table 1, the detection rates of lethal structural anomalies increased with elevated NT thickness (χ2_trend_ = 282.75, *p* < 0.05). Similarly, the incidence of cardiac ultrasound anomalies rose with increased NT thickness (χ2_trend_ = 31.81, *p* < 0.05).

**Table 1:** Summary of results for fetuses with increased NT thickness.

NT (mm)	iNT-alone (%)	iNT + SM/SA(%)	iNT + sSA(%)	Cardiac ultrasound anomaly		Total
				(+)	(−)	
2.5–2.9	239 (81.6)	42 (14.3)	12 (4.1)	14 (4.8)	279 (95.2)	293 (14.5)
3.0–3.4	470 (84.2)	63 (11.3)	25 (4.5)	27 (4.8)	531 (95.2)	558 (27.8)
3.5–4.4	462 (76.8)	108 (17.9)	32 (5.3)	46 (7.6)	556 (92.4)	602 (30.0)
4.5–5.4	171 (67.3)	51 (20.1)	32 (12.6)	26 (10.2)	228 (89.8)	254 (12.6)
5.5–6.4	70 (54.7)	28 (21.9)	30 (23.4)	15 (11.7)	113 (88.3)	128 (6.4)
≥6.5	58 (33.2)	20 (11.4)	97 (55.4)	29 (16.6)	146 (83.4)	175 (8.7)
Total	1,470 (73.1)	312 (15.5)	228 (11.4)	157 (7.8)	1853 (92.2)	2010

NT, nuchal translucency; iNT, increased nuchal translucency; SM, soft mark; SA, structural abnormalities; sSA, severe structural abnormalities; (+), with cardiac ultrasound anomaly; (−), without cardiac ultrasound anomaly.

### The relationship between NT thickness and chromosomal abnormalities detection

The detection rates of numerical chromosomal abnormalities were 15.4% (309/2,010) and 17.3% (297/1,717) in the NT ≥2.5 mm and ≥ 3.0 mm groups, respectively. Notably, the incidence of numerical chromosomal abnormalities in the NT increased group was significantly higher than that in the normal NT combined with a single soft mark group (15.4% vs. 0.8%, χ2 = 41.86, *p* < 0.05). The ratio of pathogenic/likely pathogenic CNVs also increased (5.5% vs. 1.1%, χ2 = 9.32, *p* < 0.05). With increasing NT thickness, the incidence of numerical chromosomal abnormalities significantly increased (χ2_trend_ = 275.11, *p* < 0.05). The detection ratio of pathogenic/likely pathogenic CNVs exhibited an increasing trend (χ2_trend_ = 8.60, *p* < 0.05), with the highest detection ratio found in the NT 4.5–5.4 mm group (8.7%). Trisomy 21 was prevalent in fetuses with NT thickness less than 4.5 mm (65.6%, 80/122), while trisomy 18 was more common in fetuses with NT thickness greater than 4.5 mm (69.3%, 43/62), and X monosomy was predominant in fetuses with NT thickness 5.5 mm or more (82.4%, 61/74), as shown in Table 2.

**Table 2:** Identification of CNVs in different groups of NT measurement.

NT (mm)	Numerical abnormalities of chromosomes						P/LP CNVs	VUS CNVs	Normal	Total
	Total	Trisomy-21	Trisomy-18	Trisomy-13	monosomy-X	Other				
<2.5	2 (0.8)	0 (0.0)	1 (0.4)	0 (0.0)	0 (0.0)	1 (0.4)	3 (1.1)	20 (7.6)	237 (90.5)	262
2.5–2.9	12 (4.1)	8 (2.7)	1 (0.3)	1 (0.3)	0 (0.0)	2 (0.7)	15 (5.1)	24 (8.2)	242 (82.6)	293
3.0–3.4	42 (7.5)	23 (4.1)	7 (1.3)	4 (0.7)	3 (0.5)	5 (0.9)	29 (5.2)	30 (5.4)	457 (81.9)	558
3.5–4.4	82 (13.6)	49 (8.1)	10 (1.7)	6 (1.0)	5 (0.8)	12 (2.0)	26 (4.3)	43 (7.1)	451 (74.9)	602
4.5–5.4	42 (16.5)	19 (7.5)	9 (3.5)	4 (1.6)	5 (2.0)	5 (2.0)	22 (8.7)	16 (6.3)	174 (68.5)	254
5.5–6.4	41 (32.0)	14 (10.9)	7 (5.5)	4 (3.1)	10 (7.8)	6 (4.7)	6 (4.7)	6 (4.7)	75 (58.6)	128
≥6.5	90 (51.4)	9 (5.1)	27 (15.4)	2 (1.1)	51 (29.1)	1 (0.6)	13 (7.4)	6 (3.4)	66 (37.7)	175
Total	311 (13.7)	122 (5.4)	62 (2.7)	21 (0.9)	74 (3.3)	32 (1.4)	114 (5.0)	145 (6.4)	1702 (74.9)	2,272
χ2 _trend_	275.11	22.33	84.00	–	217.58	–	8.60	3.35	184.84	
*p*-value	<0.0001	<0.0001	<0.0001	–	<0.0001	–	<0.05	>0.05	<0.0001	

P/LP CNVs, pathogenic/likely pathogenic copy number variations; VUS CNV, variant of uncertain significance copy number variations.

### Comparison of chromosomal abnormalities in different ultrasound abnormal manifestation groups

The median NT thicknesses for Groups A, B, C, and D were 1.3 mm, 3.5 mm, 3.8 mm, and 5.8 mm, respectively. The incidence of numerical chromosomal abnormalities and pathogenic/likely pathogenic CNVs increased correspondingly with the severity of ultrasound manifestations (Table 3). Trisomy 21 was mostly detected in Group B (59.0%, 72/122), while trisomy 18 (46.8%, 29/62), trisomy 13 (61.9%, 13/21), and X monosomy (60.8%, 45/74) were prevalent in Group D. Lethal structural abnormalities such as hydrops fetalis (126 cases), severe congenital heart disease (48 cases), and craniocerebral dysplasia (26 cases) were associated with high rates of aneuploidy 61.1% (77/126), 27.1% (13/48), and 42.3% (11/26) and pathogenic/likely pathogenic CNVs 7.1% (9/126), 12.5% (6/48), and 23.1% (6/26), respectively. Trisomy 18 (18.3%, 23/126) and X monosomy (34.9%, 44/126) were more common in hydrops fetalis, while trisomy 13 (42.3%, 11/26) was more common in craniocerebral dysplasia.

**Table 3:** Identification of CNVs and aneuploidies in different ultrasound abnormal manifestation groups.

Group	Numerical abnormalities of chromosomes						P/LP CNVs	VUS CNVs	Normal	Total
	Total	Trisomy-21	Trisomy-18	Trisomy-13	Monosomy-X	Other				
A	2 (0.8)	0 (0.0)	1 (0.4)	0 (0.0)	0 (0.0)	1 (0.4)	3 (1.1)	20 (7.6)	237 (90.5)	262
B	136 (9.3)	72 (4.9)	15 (1.0)	7 (0.5)	23 (1.6)	19 (1.3)	70 (4.8)	94 (6.4)	1,170 (79.6)	1,470
C	74 (23.7)	42 (13.5)	17 (5.4)	1 (0.3)	6 (1.9)	8 (2.6)	21 (6.7)	21 (6.7)	196 (62.8)	312
D	99 (43.4)	8 (3.5)	29 (12.7)	13 (5.7)	45 (19.7)	4 (1.8)	20 (8.8)	10 (4.4)	99 (43.4)	228
Total	311 (13.7)	122 (5.4)	62 (2.7)	21 (0.9)	74 (3.3)	32 (1.4)	114 (5.0)	145 (6.4)	1702 (74.9)	2,272
χ2 _trend_	248.24	13.61	99.35	–	146.06	–	16.24	1.55	191.96	
*p*-value	<0.0001	<0.0001	<0.0001	–	<0.0001	–	<0.0001	>0.05	<0.0001	

### Chromosomal abnormalities detection in NT from 2.5 mm to 2.9 mm group

The detection rate of pathogenic variants in the NT 2.5–2.9 mm group (group B) was more than three times that of the NT <2.5 mm group (group A) (6.7% vs. 1.9%, OR = 3.6). While, this incidence exhibited a 5.7-fold increase in the NT 3.0–3.4 mm group (10.0% vs. 1.9%, OR = 5.7). However, no significant difference was observed between NT 2.5–2.9 mm and NT 3.0–3.4 mm in group B. Our data suggest that a nuchal thickness of 2.5 mm might represent a critical threshold for risk stratification. Additionally, the presence of other soft markers and structural anomalies proved to be a valuable indicator in the prenatal setting. Once increased NT was combined with soft markers/structural anomalies, the incidence of genomic aberrations increased by 12.1 times, surpassing even the rate observed in the NT 3.5–4.4 mm of Group B. More details are presented in Table 4.

**Table 4:** Comparison of the detection risk of pathogenic variations in NT thickness < 4.5 mm fetuses.

Groups	Pathogenic variations			χ2^a^	*p*-value^a^	OR^a^(95%CI)	χ2^b^	*p*-value^b^	OR^b^(95%CI)	χ2^c^	*p*-value^c^	OR^c^(95%CI)	Cases
	Aneuploidy	P/LP CNVs	Total										
A	2 (0.8)	3 (1.1)	5 (1.9)	Ref.			7.129	<0.05	0.3 (0.1 ~ 0.7)	16.690	<0.0001	0.2 (0.07 ~ 0.4)	262
B													
2.5–2.9	5 (2.1)	11 (4.6)	16 (6.7)	7.129	<0.05	3.6 (1.3 ~ 10.2)	Ref.			2.138	>0.05	0.6 (0.4 ~ 1.2)	239
3.0–3.4	25 (5.3)	22 (4.7)	47 (10.0)	16.690	<0.0001	5.7 (2.2 ~ 14.5)	2.138	>0.05	1.5 (0.9 ~ 2.8)	Ref.			470
3.5–4.4	45 (9.7)	15 (3.2)	60 (13.0)	25.110	<0.0001	7.7 (3.0 ~ 19.4)	6.452	<0.05	2.1 (1.2 ~ 3.7)	2.045	>0.05	1.3 (0.9 ~ 2.0)	462
C													
2.5–2.9	6 (14.3)	2 (4.8)	8 (19.0)	21.958	<0.0001	12.1 (3.7 ~ 39.1)	6.978	<0.05	3.3 (1.3 ~ 8.2)	3.292	>0.05	2.1 (0.9 ~ 4.8)	42
3.0–3.4	13 (20.6)	3 (4.8)	16 (25.4)	42.555	<0.0001	17.5 (6.1 ~ 50.1)	18.409	<0.0001	4.7 (2.2 ~ 10.1)	12.635	<0.0001	3.1 (1.6 ~ 5.8)	63
3.5–4.4	27 (25.0)	7 (6.5)	34 (31.5)	70.930	<0.0001	23.6 (8.9 ~ 62.5)	37.057	<0.0001	6.4 (3.3 ~ 12.3)	33.631	<0.0001	4.1 (2.5 ~ 6.8)	108
D													
2.5–2.9	1 (8.3)	2 (16.7)	3 (25.0)	14.208	<0.0001	17.1 (3.5 ~ 83.0)	5.472	<0.05	4.6 (1.1 ~ 18.8)	2.832	>0.05	3.0 (0.8 ~ 11.5)	12
3.0–3.4	4 (16.0)	4 (16.0)	8 (32.0)	41.083	<0.0001	24.2 (7.1 ~ 82.0)	17.537	<0.0001	6.6 (2.5 ~ 17.5)	11.633	<0.0001	4.2 (1.7 ~ 10.3)	25
3.5–4.4	10 (31.3)	4 (12.5)	14 (43.8)	75.814	<0.0001	40.0 (12.9 ~ 123.4)	39.363	<0.0001	10.8 (4.5 ~ 25.7)	31.969	<0.0001	7.0 (3.3 ~ 15.0)	32

a The detection rate of chromosomal abnormalities in group A was used as a control.

b The detection rate of chromosomal abnormalities in the group B of NT 2.5–2.9 mm was used as a control.

c The detection rate of chromosomal abnormalities in the group B of NT 3.0–3.4 mm was used as a control.

### Pregnancy outcomes

Out of 2,272 pregnant women, 1,898 underwent successful follow-ups (83.5%), and the outcomes were detailed in Table 5. Termination of pregnancy or stillbirth occurred in 561 fetuses, with 309 (55.1%) exhibiting chromosomal numerical abnormalities, 76 fetuses showing pathogenic/likely pathogenic CNVs (13.5%), 18 fetuses with variants of uncertain significance (VUS) (3.2%), and 113 fetuses presenting lethal or non-lethal structural abnormalities of unknown genetic etiology (20.1%). Additionally, nine fetuses were terminated due to concerns related to increased NT, eight fetuses resulted in *in utero* death 2 weeks post chorionic villi sampling, five fetuses experienced *in utero* death in late pregnancy with unknown reasons, five fetuses had monogenic disorders, two fetuses carried likely benign CNVs, one fetus experienced *in utero* death due to umbilical cord knotting, one fetus was attributed to maternal hyperemesis gravidarum, and 14 fetuses were terminated for other reasons.

**Table 5:** Comparison of birth outcomes in each group.

Group	Live birth	Termination/stillbirth	Total
NT (mm)			
<2.5	203 (96.7)	7 (3.3)	210
2.5–2.9	220 (88.4)	29 (11.6)	249
3.0–3.4	376 (80.3)	92 (19.7)	468
3.5–4.4	357 (73.3)	130 (26.7)	487
4.5–5.4	124 (57.7)	91 (42.3)	215
5.5–6.4	36 (33.6)	71 (66.4)	107
≥6.5	21 (13.0)	141 (87.0)	162
χ2	433.90		
*p*-value	<0.0001		
Feature of ultrasound			
A	203 (96.7)	7 (3.3)	210
B	973 (82.0)	214 (18.0)	1,187
C	156 (56.9)	118 (43.1)	274
D	5 (2.2)	222 (97.8)	227
Total	1,337 (70.4)	561 (29.6)	1898
χ2	619.96		
*p*-value	<0.0001		

Regarding live births, with a median follow-up age of 16 months, three infants died in the neonatal period—one with trisomy X, one with Tetralogy of Fallot, and one with the 22q11.21 microduplication. A 9-month-old infant with atrial septal defect succumbed to COVID-19. The incidence of neurodevelopmental delay showed no significant difference between the increased NT group and the normal NT combined with a single soft mark group in live births (0.97% vs. 0.49%, χ2 = 0.065, *p* > 0.05) when evaluating Gesell Developmental Schedules scores below 75 after 6 months of age.

## Discussion

This retrospective study comprehensively reviewed 2,272 pregnancies, investigating the intricate relationships among genomic aberrations, pregnancy outcomes, and NT thickness. Increased NT thickness was strongly associated with chromosomal abnormalities, revealing an identification rate of 15.4% (309/2,010) using a 2.5 mm cutoff of and 17.3% (297/1,717) using a 3.0 mm cutoff of, consistent with the literature (11), albeit lower than the 22.76% reported by Mastromoro et al. (12), likely due to the exclusion of age-related risks. Combining CMA or CNV-seq with NT screening enhanced the detection of genomic aberrations in these pregnancies, improving the yield by 1.8 ~ 5.5% compared to previous studies (4, 12–14).

The present data indicated that most trisomy 21 fetuses had an NT thickness below 4.5 mm, while trisomy 18 fetuses predominantly exhibited NT thicknesses above 4.5 mm, consistent with the literature (15). During comparison of groups A, B, C, and D, 59.0% of trisomy 21 cases were predominantly found in group B, while most trisomy 18, trisomy 13, and X monosomy fetuses were identified in group D. Our results further indicate that the risk of chromosomal abnormalities in fetuses with increased NT and ultrasonographic anomalies was significantly higher than that in fetuses with isolated increased NT. Numerical chromosomal abnormalities emerged as the primary contributors to lethal structural abnormalities such as hydrops fetalis, severe congenital heart disease, and craniocerebral dysplasia. Additionally, P/LP CNVs were implicated in a substantial proportion of severe congenital heart disease and craniocerebral dysplasia cases. Our findings support the association between cardiac defects and increased NT, which is in accordance with the literature (16). This positive correlation between NT thickness and cardiac ultrasound anomalies was validated in our study, with the detection rate of cardiac anomalies escalating from 7.6% in the NT 3.5–4.4 mm group to 16.6% in the NT ≥ 6.5 mm group. Thus, our data provide crucial insights into the associations between trisomy, NT thickness, and significant structural anomalies, enhancing existing clinical correlations.

Currently, there is a lack of consensus in international guidelines regarding the NT value cutoff, with commonly used thresholds being 3.0 mm, 3.5 mm, or the 99th percentile (1–3), which contributes to the anxiety experienced by pregnant women and poses challenges for clinical management and genetic counseling, particularly for fetuses with NT measurements between 2.5 mm and 2.9 mm. Within the specific cohort presenting with a NT measurement between 2.5 and 2.9 mm, our study identified chromosomal abnormalities in 9.2% of cases. This finding aligns with previously published data, albeit with some variability. For example, Zhang et al., reported a detection rate of 8.2% using the CMA platform (4). Similarly, a tertiary care center in China observed a rate of 6.2% in fetuses with NT ranging from the 95th percentile to 3.0 mm (7). However, other studies documented lower rates, such as 5.1% reported by Yin et al. (9), while Wang et al. noted a higher rate of 12.7% (8). A recent systematic meta-analysis by Mastromoro et al., reported a range of 8.4 to 13.0% for the chromosomal abnormality detection rate in the NT 2.5–2.9 mm group, compared to 6.6% ~ 33.8% for the NT 3.0–3.4 mm group (12). Interestingly, some studies documented even higher rates in the 2.5–2.9 mm group than the 3.0–3.4 mm group (8.2% versus 6.9%) (4), highlighting its potential clinical significance. Further underscoring this importance, our study found fetal congenital defects in 18.4% of fetuses with NT 2.5–2.9 mm. Fantasia et al. (17) also recognized the significance of mild NT elevations (between the 95th and 99th percentiles), reporting rates of 12.1% for chromosomal abnormalities and 13.7% for congenital defects. However, it’s important to note that an NT of 3.0 mm does not directly correlate to the 95th percentile. As demonstrated by Hui et al.’s fetal NT distribution (18), the 95th percentile varies depending on crown-rump length (CRL), ranging from 2.1 mm at CRL 45 mm to 3.3 mm at CRL 84 mm. This emphasizes the importance of NT measurement must also be considered in relation to the measurement of CRL. Detection rates varied across studies, possibly influenced by sample size and population differences. Additionally, our study found no significant difference in the detection rates of chromosomal abnormalities between cases with an isolated increased NT of 2.5–2.9 mm (6.7%, 16/239) and cases with an isolated increased NT of 3.0–3.4 mm (10.0%, 47/470). Taken together, NT measurements between 2.5 mm and 2.9 mm are associated with an elevated risk of fetal chromosomal abnormalities and congenital defects.

Presently, noninvasive prenatal testing is widely recognized as an accurate method for detecting common aneuploidies. Due to the elevated risk of fetal loss associated with invasive prenatal diagnosis, pregnant individuals in China, particularly those with isolated NT measurements ranging from 2.5 to 2.9 mm, often opt for NIPT (19). However, it should be borne in mind that NIPT may miss some atypical chromosomal abnormalities (ACA). Miranda et al. (20) reported missed diagnosis rates of 12 and 19% for chromosomal abnormalities in fetuses with NT > 99th using targeted NIPT and extended NIPT (including sex chromosomes), respectively. The missed diagnoses were primarily related to sex chromosomal abnormalities, pathogenic CNVs, and Noonan syndrome (20). While NIPT-Plus with high depth of coverage can detect chromosome deletion/duplication syndromes and some non-syndromic CNVs (21), Xie et al. found a 4.7% (1/21) failure rate in detecting fetuses with chromosomal aneuploidy, and three out of five fetuses with pathogenic CNVs were missed using NIPT-Plus (19). Despite the incremental detection rate of only 1.32% for CMA in the subgroup of apparently isolated NT 2.5–2.9 mm fetuses as reported by Mastromoro et al. (12), our study revealed a higher rate of 4.6% pathogenic/likely pathogenic CNVs in fetuses with isolated NT measurements within the same range. Furthermore, when soft markers or structural anomalies were present in combination with increased NT measurements, the detection rate increased to 7.4% (4/54) in our study. The presence of additional anomalies appears to increase the likelihood of identifying pathogenic/likely pathogenic CNVs. Additionally, Fantasia et al. reported that ACA was exclusively observed in fetuses with congenital malformations (17). Some studies suggested that utilizing a dynamic cutoff value of 1.9 MoM or the 99th centile might enhance the screening efficiency for atypical chromosomal abnormalities (3). Therefore, NIPT may be considered for fetuses with isolated increased NT of 2.5–2.9 mm, especially for those who decline to have invasive prenatal diagnosis. But it should be pointed out that, comprehensive prenatal counseling and systematic ultrasound examinations are important in this scenario. When soft markers/structural anomalies are present, NIPT may not be a potent option and should not be recommended.

In our study, after excluding cases attributed to pathogenic chromosomal abnormalities and fetal structural abnormalities, we found a significant number of pregnancy losses, amounting to 34.5% (20/58), were linked to reports of CNVs of unknown significance or likely benign CNVs. This finding has raised concerns within our research team regarding the reporting of CNVs, emphasizing the critical role of genetic counseling in clinical practice. The primary issue in reporting CNVs of unknown significance or likely benign CNVs is potential anxieties, personal emotions, coupled with broader social and ethical implications. Based on recommendations outlined by Armour et al. (1), it is suggested that only submicroscopic duplications longer than 1 Mb or deletions longer than 500 Kb should be reported. Implementing this recommendation will benefit some families by halving the number of cases reported, and easing the workload of genetic counseling in clinics.

This study conducted a comprehensive analysis of chromosomal abnormalities and pregnancy outcomes in fetuses with varying NT thicknesses, highlighting the significance of mild NT elevations (2.5 mm to 2.9 mm), and providing valuable data for improving the prenatal diagnosis and evaluation strategy for fetuses with increased NT. However, there are several potential limitations in this study. Firstly, it relied on retrospective data collection. Secondly, the incidence of structural defects in fetuses with NT measurements between 2.5–2.9 mm was higher than previously reported in the literature (7, 17). This discrepancy may be attributed to the fact that some pregnant women opted for NIPT instead of invasive prenatal diagnosis, potentially influencing the observed incidence of structural defects. In addition, some pregnancies with increased NT thickness may be attributed to single gene defects, such as Noonan syndrome, Cornelia Lange syndrome, Smith-Lemli-Opitz syndrome, and multiple skeletal disorders (22–24), in which cases panel sequencing or Whole exome sequencing (WES) may be more helpful (25).

## Conclusion

This study underscores the significance of CNV analysis in identifying additional clinically relevant pathogenic or likely pathogenic CNVs in fetuses with increased NT. Specifically, it revealed a substantial increase of 5.5% in the detection of such CNVs. It is crucial to exercise caution and be careful for fetuses with NT values between 2.5 and 2.9 mm, as our findings indicate a noteworthy 18.4% incidence of structural defects and a 9.2% prevalence of pathogenic chromosomal abnormalities within this range. Thus, it is important to conduct thorough prenatal counseling and systematic ultrasound examinations to ensure comprehensive evaluation and understanding of potential risks. Importantly, NIPT should not be recommended for fetuses with NT values of 2.5–2.9 mm when combined with soft markers or structural anomalies.

## Data availability statement

The original contributions presented in the study are included in the article/supplementary material, further inquiries can be directed to the corresponding author.

## Ethics statement

The studies involving humans were approved by Ethics Committee of Henan Provincial People’s Hospital (2021-No.171). Prior to participation, all individuals involved provided informed consent and underwent genetic counseling.

## Author contributions

JH: Data curation, Formal analysis, Writing – original draft. DW: Data curation, Formal analysis, Writing – review & editing. J-HH: Data curation, Formal analysis, Investigation, Writing – review & editing. J-YW: Data curation, Investigation, Writing – review & editing. XL: Data curation, Investigation, Writing – review & editing. Z-YW: Formal analysis, Writing – review & editing. YW: Funding acquisition, Investigation, Resources, Writing – review & editing. H-YL: Investigation, Resources, Supervision, Writing – review & editing.
